# A new species of *Bembidion* Latrielle 1802 from the Ozarks, with a review of the North American species of subgenus *Trichoplataphus* Netolitzky 1914 (Coleoptera, Carabidae, Bembidiini)

**DOI:** 10.3897/zookeys.147.1872

**Published:** 2011-11-16

**Authors:** Drew A. Hildebrandt, David R. Maddison

**Affiliations:** 1 710 Laney Road, Clinton, MS 39056 USA; 2 Department of Zoology, Oregon State University, Corvallis, OR 97331, USA

**Keywords:** *Bembidion*, Bembidiini, Trechinae, COI, 28S rDNA, taxonomy, systematics

## Abstract

A new species of *Bembidion (Trichoplataphus* Netolitzky) from the Ozark Plateau of Missouri and Arkansas is described (*Bembidion ozarkense* Maddison and Hildebrandt). It is distinguishable from the closely related species, *Bembidion rolandi* Fall, by characteristics of the male genitalia, and sequences of the genes cytochrome oxidase I and 28S ribosomal DNA. A brief review of the North American species of *Trichoplataphus* is presented, including a key to species.

## Introduction

While identifying beetles of the genus *Bembidion* Latreille that the senior author collected in Arkansas and Missouri some years ago, he came across a series of specimens of subgenus *Trichoplataphus* Netolitzky that keyed to *Bembidion rolandi* Fall using the latest revision of the group (Lindroth 1963). However, the collecting locality was far west of the known range of that species (Bousquet and Larochelle 1993). He contacted the junior author for his opinion on the species identification, which initiated a study that has resulted in the present work.

The purpose of this paper is to describe the new species that was discovered and provide a review of the North American species of the subgenus *Trichoplataphus*. The most recent revision of the subgenus was included in Lindroth’s treatment of the genus for the northern U.S. and Canada (Lindroth 1963), and this paper builds on that work by addition of the new species, and modification of Lindroth’s key.

It is with great pleasure that we dedicate this paper to Dr. Ross Bell. For one of us he has been a very generous, informative and helpful correspondent, for the other a mentor and close colleague. Both of us have benefited immeasurably by our interactions with him. This paper is a small contribution to the fauna of an area that has interested Ross since his youth, when he first collected the endemic *Chlaenius viduus* Horn in the Ozarks.

## Methods

Approximately 580 specimens of *Bembidion (Trichoplataphus)* were examined as part of this review; no effort was made to do a complete survey of specimens in existing collections. Specimens were examined from the following collections; each collection listing begins with the codon used in the text.

CMNH Carnegie Museum of Natural History, Pittsburgh, PA, USA (Robert L. Davidson)

DAHC Drew A. Hildebrandt collection, Clinton, MS. USA

OSAC Oregon State Arthropod Collection, Oregon State University, Corvallis, OR (David R. Maddison)

TAMU Texas A&M University, College Station, TX, USA (Edward C. Riley)

**Collecting methods.** Specimens were collected by hand, by splashing or pouring water on gravel bars or by raking the gravel by hand during the day to dislodge the beetles hiding under the gravel; or at night when the beetles were out, actively moving about on the surface. During the day, raking the gravel by hand works best for collecting specimens of this subgenus because of their behavior when disturbed. Unlike members of most other Bembidiini, individuals of *Trichoplataphus* tend to run under the water and cling to stones, and thus they are more difficult to catch (Davidson 1978).

Specimens for morphological studies were killed and preserved in sawdust or woodchips to which ethyl acetate was added. Specimens for DNA sequencing were collected into 95% or 100% ethanol, with best results obtained if the abdomen was slightly separated from the rest of the body to allow better penetration.

**Morphological methods**. Methods for studying adult structures, and terms used, are given in Maddison (1993).

All measurements were made on dried, pinned or pointed specimens using an Olympus SZ6045 zoom stereo microscope with a calibrated ocular micrometer. Character examinations were made at magnifications ranging from 10x to 63x; all measurements were made at 30x. Standardized body length (SBL) was measured following the protocol of Kavanaugh (1979).

**Taxon sampling for DNA studies.** We sequenced DNA from 18 specimens of *Bembidion (Trichoplataphus)*, including all known North American species, as well as *Bembidion mimekara* Toledano and Schmidt from China ((Table 1)). Preliminary analyses of multiple genes across *Bembidion* (Maddison, unpublished) indicate that the sampled *Trichoplataphus* form a clade, and that, among the species sampled*, B. mimekara* is the sister group to the North American species. DNA vouchers are housed in the David Maddison voucher collection at Oregon State University.

**DNA sequencing.** Methods for obtaining DNA sequences are described in Maddison (2008). In brief, we obtained ca. 1000 bases of sequence data in the D1 through D3 domains of 28S ribosomal DNA (28S or 28S rDNA) and 650 to 750 bases of cytochrome oxidase I (COI). Fragments for these genes were amplified using the Polymerase Chain Reaction on an Eppendorf Mastercycler Thermal Cycler, using either Eppendorf Hotmaster Taq or TaKaRa Ex Taq and the basic protocols recommended by the manufacturers. Primers and details of the cycling reactions used are given in Maddison (2008). In particular, we used the primer pair LS58F and LS998R and the pair NLF184/21 and LS1041R to amplify and sequence 28S rDNA. For COI, two amplification and sequencing strategies were used: use of primer pairs B1490 and Bcoi2R (see Maddison 2008), or the LCO1490 and HCO2198 primers (Hebert et al. 2003). Amplified products were cleaned, quantified, and sequenced at the University of Arizona’s Genomic and Technology Core Facility using either a 3730 or 3730 XL Applied Biosystems automatic sequencer.

Assembly of multiple chromatograms for each gene fragment and initial base calls were made with Phred (Green and Ewing 2002) and Phrap (Green 1999) as orchestrated by Mesquite’s Chromaseq package (Maddison and Maddison 2009a; Maddison and Maddison 2009b), with subsequent modifications by Chromaseq and manual inspection. Multiple peaks at a single position in both reads were coded using IUPAC ambiguity codes.

Sequences have been deposited in GenBank with accession numbers JF800039 through JF800074.

**Alignment.** Alignment for both genes could be unambiguously determined, as there were no insertion or deletion events evident in the COI sequences, and only one in 28S, in which *Bembidion mimekara* has 2 bases not present in the other species.

**Molecular phylogenetic analysis.** Models of nucleotide evolution were chosen with the aid of ModelTest version 3.7 (Posada 2005). For 28S rDNA, the model chosen by the Akaike Information Criterion (AIC) was a General Time Reversible (GTR) rate matrix with a proportion of sites being invariant (the GTR + I model); for COI it was a GTR rate matrix with site variation following a gamma distribution (the GTR + G model).

Likelihood analyses of nucleotide data were conducted using Garli version 1.0.699 (Zwickl 2006). For each matrix, 1000 non-parametric bootstrap search replicates were conducted.

### Results from molecular analyses

**Figure 1.**
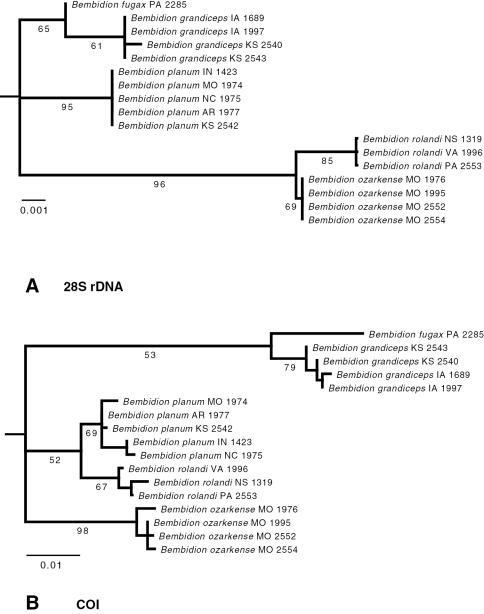
Maximum-likelihood bootstrap trees for **A** 28S rDNA and **B** COI. Each terminal taxon is a single specimen, whose state or province of origin is shown, using standard abbreviations, after the species name; the four digits at the end of the name form the voucher number. Numbers below each branch are the percentage of bootstrap replicates showing that branch; values are not shown within each species. Branch lengths are proportional to number of substitutions per site, as reconstructed by Garli. *Bembidion (Trichoplataphus) mimekara*, the outgroup, is not shown because of its long branch length.

Specimens of each of the species have distinctive COI and 28S sequences (Fig. 1). *Bembidion rolandi* shows consistent differences from similar specimens from the Ozarks, which we are describing as *Bembidion ozarkense*. All specimens of *Bembidion rolandi* differ from all specimens of *Bembidion ozarkense* at 18 bases among the 766 sequenced sites in COI (2.3% divergent), as well as 2 of the 995 sites sampled of 28S rDNA (0.2%). These two species are reciprocally monophyletic in the inferred phylogenetic trees (Fig. 1).

**Table 1.** *Bembidion (Trichoplataphus)* specimens sampled for DNA sequences. Four-digit numbers under “#” are D.R. Maddison DNA voucher numbers. The specimen marked with a “*” is the holotype of *Bembidion ozarkense*, n.sp.

Species	#	Locality
*Bembidion mimekara* Toledano & Schmidt	1366, 2448	China: Yunnan: Gongshan County, Dulongjiang Township, Kongdan, east bank of Dulong Jiang. 1510 m, 27.87764°N, 098.33618°E
*Bembidion planum* (Haldeman)	1977	USA: Arkansas: Henderson Creek at route 23, Ozark Mountains
*Bembidion planum* (Haldeman)	1423	USA: Indiana: Crawford Co., English, Camp Fork Creek, 150m 38.3334°N, 86.4646°W
*Bembidion planum* (Haldeman)	2542	USA: Kansas: Jefferson Co., Little Slough Ck, W of Kiowa Rd. 39.23355°N, 95.39497°W
*Bembidion planum* (Haldeman)	1974	USA: Missouri: Washington Co., Big River at route 21, 245m, 37.8121°N, 90.7723°W
*Bembidion planum* (Haldeman)	1975	USA: North Carolina: Mitchell Co., Penland, North Toe River, 744m, 35.9293°N, 82.1149°W
*Bembidion fugax* (LeConte)	2285	USA: Pennsylvania: Perry Co., Susquehanna River 5 km N New Buffalo, 40.4909°N, 76.9535°W
*Bembidion grandiceps* Hayward	1689	USA: Iowa: Webster Co., Des Moines River near Stratford, 275m, 42.3101°N, 93.9371°W
*Bembidion grandiceps* Hayward	1997	USA: Iowa: Webster Co., Des Moines River near Stratford, 275m, 42.3101°N, 93.9371°W
*Bembidion grandiceps* Hayward	2540	USA: Kansas: Jefferson Co., Little Slough Ck, W of Kiowa Rd. 39.23355°N, 95.39497°W
*Bembidion grandiceps* Hayward	2543	USA: Kansas: Jefferson Co., Little Slough Ck, W of Kiowa Rd. 39.23355°N, 95.39497°W
*Bembidion ozarkense*, sp. n.	1976	USA: Missouri: Carter Co., Current River at Van Buren, 135m, 36.9904°N, 91.0100°W
*Bembidion ozarkense*, sp. n.	1995	USA: Missouri: Washington Co., Irondale, Big River, 230m, 37.8302°N, 90.6895°W
*Bembidion ozarkense*, sp. n.	2552*	USA: Missouri: Carter Co., Current River at Van Buren, 135m, 36.9904°N, 91.0100°W
*Bembidion ozarkense*, sp. n.	2554	USA: Missouri: Carter Co., Current River at Van Buren, 135m, 36.9904°N, 91.0100°W
*Bembidion rolandi* Fall	1319	Canada: Nova Scotia: Economy River at route 2, 45.3868°N, 63.8992°W
*Bembidion rolandi* Fall	1996	USA: Virginia: Rockbridge Co., Maury River, Glasgow, 215m, 37.6329°N, 79.4431°W
*Bembidion rolandi* Fall	2553	USA: Pennsylvania: Perry Co., Susquehanna River 5 km N New Buffalo, 40.4909°N, 76.9535°W

## Descriptions and identification of taxa

### Subgenus *Trichoplataphus* Netolitzky, 1914

The subgenus *Trichoplataphus* of the genus *Bembidion* (Coleoptera: Carabidae: Trechinae: Bembidiini) contains 19 described species in the Palaearctic Region (Toledano and Schmidt 2010), and four species reported from North America (Bousquet and Larochelle 1993; Lindroth 1963; Lorenz 2005).

Adult specimens of the subgenus are easily separated from most other groups in North America by the irregular scattering of setiferous punctures on the abdominal sterna (Lindroth 1963). The only other species in North America with adults that have extra setiferous punctures on the abdominal sterna is *Bembidion hasti* Sahlberg, in which the setae are arranged in a regular, transverse row on each sternum.

**Figure 2.**
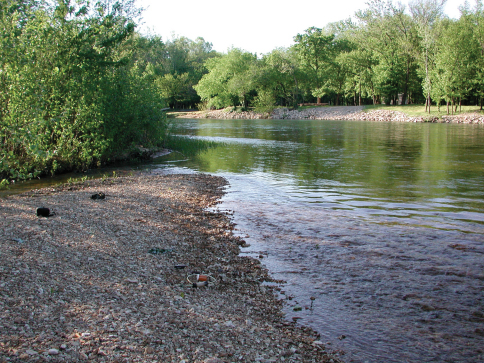
Type locality of *Bembidion ozarkense*, the Current River at Van Buren (USA: Missouri: Carter County).

Members of *Trichoplataphus* are found on open, bare gravel bars and banks, sometimes mixed with clay or sand (Fig. 2; Larochelle and Larivière 2003). Although mostly confined to running waters, they have also been collected among gravel and pebbles on banks of islands in Lake Champlain (Davidson 1978). These beetles are macropterous and have been found to fly to light (Larochelle and Larivière 2003).

The North American species of *Trichoplataphus* are:

*Bembidion ozarkense* sp. n.

*Bembidion rolandi* Fall, 1922

*Bembidion grandiceps* Hayward, 1897

*Bembidion fugax* (LeConte, 1848)

*Bembidion planum* (Haldeman, 1843)

### Identification of Species Using Morphological Data

Species of this subgenus are very difficult to tell apart using morphological features, as the known differences are very subtle. Future morphological studies may reveal better characters to separate them. In the meantime, the key we present below (a modified version of couplets 77 through 80 in Lindroth 1963) should help in identifying specimens to species. The reader is strongly encouraged to read carefully the descriptions in Lindroth (1963) and this paper; sometimes species can be separated on a suite of characters that in aggregate are more useful than in isolation. In the following key, “fig.” refers to figures from Lindroth (1963); “Fig.” refers to figures in this paper.

**Table:** 

77	Mentum with epilobes of normal size for a *Bembidion*, projected notably anteriad of the mentum tooth (Fig. 3A)	77a
–	Mentum with epilobes much reduced, projected about as far anteriad as the mentum tooth, and with a prominent concavity on the lateral side of each epilobe (Fig. 3B)	78
77a	From the Ozark Plateau of Missouri and Arkansas; aedeagus with tip relatively blunt, and not sharply bent downward (Fig. 4A and 4B)	*Bembidion ozarkense*
–	From east of the Mississippi River; aedeagus with tip more narrowly pointed, abruptly bent downward (Fig. 4C and 4D)	*Bembidion rolandi*
78	Prothorax strongly constricted at base (Lindroth 1963, fig. 144); although constricted, parallel-sided base of prothorax very short. Elytra quite flat at apex	*Bembidion grandiceps*
–	Prothorax less constricted. Elytra slightly sloping at extreme tip.	79
79	6th elytral stria hardly weaker than 5th behind shoulder but obliterated towards apex. Frontal furrows prolonged and strongly diverged behind posterior supra-orbital punctures	*Bembidion fugax*
–	6th elytral stria much weaker than 5th, often obliterated throughout. Frontal furrows ended just behind posterior supra-orbital puncture and little diverged	*Bembidion planum*

**Figure 3.**
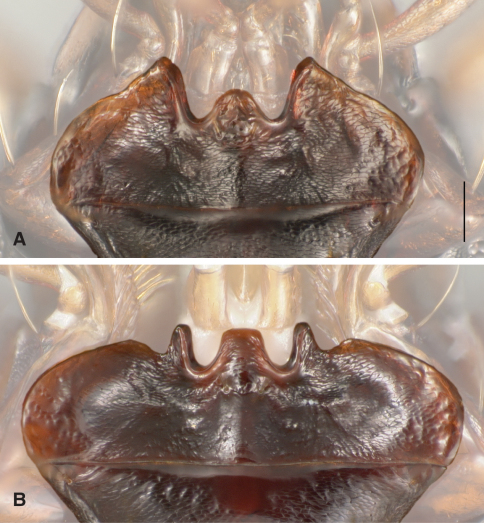
Menta of *Bembidion (Trichoplataphus)*. Scale bar is 0.1 mm. Features other than the menta have been digitally faded **A** *Bembidion ozarkense*, USA: Missouri: Carter Co., Current River at Van Buren, 135m, 36.9904°N, 91.0100°W **B** *Bembidion grandiceps*, USA: Iowa: Webster Co., Des Moines River near Stratford, 275m, 42.3101°N, 93.9371°W, voucher DNA1689.

#### Bembidion ozarkense

Maddison & Hildebrandt sp. n.

urn:lsid:zoobank.org:act:042B6160-C581-4028-BC44-421C1448C831

http://species-id.net/wiki/Bembidion_ozarkense

Fig. 3A 4A, 4B 5A 6

##### Holotype.

 Male (in OSAC), here designated, labeled “USA: Missouri: Carter Co., Current River at Van Buren, 135m, 36.9904°N, 91.0100°W, 24.iv.2005. DRM 05.015. D.R. Maddison” / “David R. Maddison DNA2552 DNA Voucher” [pale green paper] / “HOLOTYPE *Bembidion ozarkense* Maddison & Hildebrandt” [red paper]”. Genitalia in glycerine vial with specimen; extracted DNA stored separately. GenBank accession numbers for DNA sequences of the holotype are JF800056 (28S) and JF800065 (COI).

##### Paratypes.

 221 specimens as follows: USA: **Missouri**: Carter Co., Current River at Van Buren, 135m, 36.9904°N, 91.0100°W (88 specimens); Carter Co., Current River at Van Buren, 135m, 36.9924°N, 91.0167°W (3); Carter Co., Current River at Van Buren, 135m, 36.9911°N, 91.0133°W (3); Maries Co., Maries River near Argyle, 200m, 38.2700°N, 92.0007°W (10); Reynolds Co., Clark National Forest. Sutton’s Bluff Campground (6); Washington Co., Irondale, Big River, 230m. 37.8302°N, 90.6895°W (16); Washington Co., Big River at route 21, 245m. 37.8121°N, 90.7723°W (4); Washington Co., Big River at route 21, 245m, 37.8132°N, 90.7734°W (1); **Arkansas**: Crawford Co., Lee Creek (1); Marion Co., Buffalo Point St.Pk. (42); Searcy Co., 5 mi. W. Big Flat. Big Creek (47). Male genitalia have been examined from at least one specimen from each paratype locality.

Paratypes of *Bembidion ozarkense* have been deposited in the CMNH, DAHC, OSAC and TAMU, and in the collections of The Natural History Museum, London (BMNH), the California Academy of Sciences (CAS), the Field Museum of Natural History (FMNH), the Museum of Comparative Zoology (MCZ), Muséum National d’Histoire Naturelle in Paris (MNHN), the University of Arizona (UAIC), the University of Alberta Strickland Museum (UASM), and the National Museum of Natural History, Smithsonian Institution (USNM).

Eighty-two specimens examined but not designated as paratypes are: USA: **Missouri** : Pulaski Co., Devil’s Elbow. Big Piney River. 10km E. Waynesville. 734 ft. 37 50 52.4 N 92 03 42.0 W (2); **Arkansas**: Marion Co., Buffalo Point S.P. (15); Searcy Co., 5.3 mi. W. Big Flat. Big Creek. (65). The Missouri specimens are omitted from the paratype series as there are no males whose genitalia could be examined; the Arkansas specimens are omitted as they are slightly damaged.

##### Type locality.

 USA: Missouri: Carter Co., Current River at Van Buren, 135m, 36.9904°N, 91.0100°W. At the type locality (Fig. 2), adults of *Bembidion ozarkense* were common on 24 April 2005; specimens occurred in company with *Bembidion (Trichoplataphus) planum*, *Bembidion (Pseudoperyphus) antiquum* Dejean, and *Bembidion (Pseudoperyphus) chalceum* Dejean.

##### Derivation of specific epithet.

 Derived from the Ozark Plateau of Missouri and Arkansas, which encompasses the known range of this species.

##### Diagnosis.

**Figure 4.**
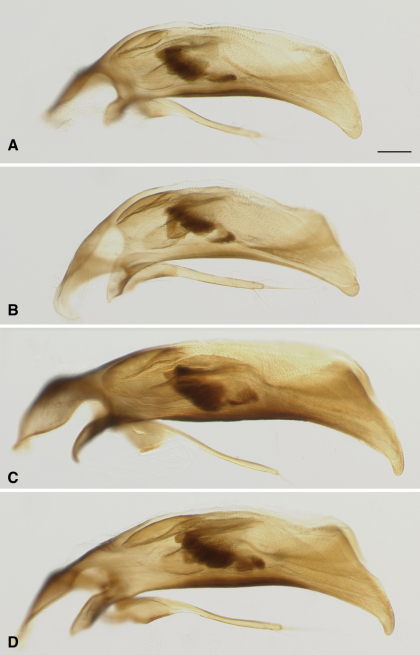
Aedeagus of male *Bembidion ozarkense* and *Bembidion rolandi*. Scale bar is 0.1 mm **A** *Bembidion ozarkense*, USA: Missouri: Carter Co., Current River at Van Buren, 135m, 36.9904°N, 91.0100°W Voucher DNA1976 **B** *Bembidion ozarkense*, USA: Missouri: Washington Co., Irondale, Big River, 230m, 37.8302°N, 90.6895°W, Voucher DNA1995 **C** *Bembidion rolandi*, USA: Virginia: Rockbridge Co., Maury River, Glasgow, 215m, 37.6329°N, 79.4431°W, Voucher DNA1996 **D** *Bembidion rolandi*, Canada: Nova Scotia: Economy River at route 2, 45.3868°N, 63.8992°W, Voucher DNA1319.

*Bembidion ozarkense* males can be recognized among North American *Trichoplataphus* by the combination of epilobes of the mentum of normal size for a *Bembidion* (Fig. 3A), and aedeagus with the tip relatively thick and not abruptly bent downward (Fig. 4A and 4B).

**Figure 5.**
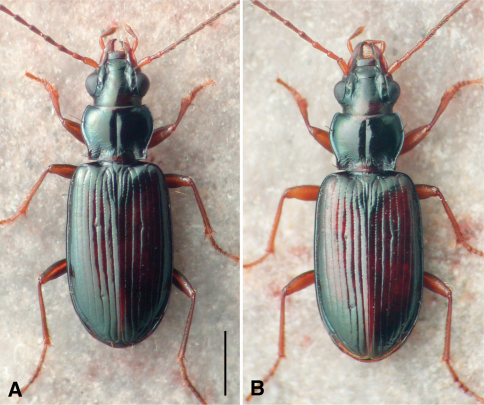
Habitus photographs. Specimens at same scale; scale bar is 1.0 mm **A** *Bembidion ozarkense*, USA: Missouri: Carter Co., Current River at Van Buren, voucher DRMV100311 **B** *Bembidion rolandi*, USA: Virginia: Rockbridge Co., Maury River at Glasgow, voucher DRMV100193.

This species is markedly similar to *Bembidion rolandi* in external form (Fig. 5), sharing with it normal mentum epilobes; narrow, flat body, with long antennae; the pronotal base notably constricted, hind angles right, with the lateral margins in front of the hind angle parallel and straight. We have not yet found any reliable external characters to separate the two species, although there are some traits by which they tend to differ. Specimens of *Bembidion ozarkense* tend to be slightly smaller (average SBL of males is 4.4 mm, of females 4.7 mm) than those of *Bembidion rolandi* (4.6 and 4.8 mm, respectively), but there is a broad range of overlap, as adults of both species range in length from 4.2 to 5.1 mm. *Bembidion ozarkense* tends to be darker than *Bembidion rolandi*, with less-rufous elytra, and with the posterior medial portion of the dorsal surface of the head darker, rarely with the rufous region of many *Bembidion rolandi*; the second antennomere of *Bembidion ozarkense* is often infuscated centrally, whereas it is usually entirely pale rufous in *Bembidion rolandi*. However, while these tendencies are noticeable in larger series, there is enough overlap between species that they are of marginal use when comparing individual specimens.

The only morphological characteristic that we have found to reliably distinguish the two species is in the male aedeagus: the tip of *Bembidion rolandi* is thin, and abruptly bent downward (Fig. 4C and 4D; n=8), traits not found in *Bembidion ozarkense* (Fig. 4A and 4B; n=30). DNA sequence data can also be definitively used to identify the species; either COI or 28S rDNA will suffice.

However, the known distribution ranges are distinctly separate, and can be used in the absence of male genitalia or DNA sequences to identify specimens.

##### Geographic distribution.

**Figure 6.**
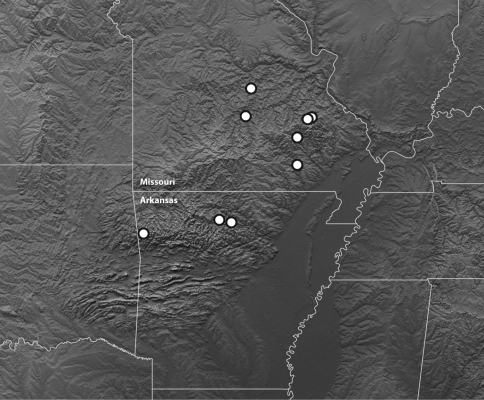
Map of the Ozark Plateau and surrounding regions showing the known distribution of *Bembidion ozarkense*. Topographic image modified from NASA/JPL, http://photojournal.jpl.nasa.gov/catalog/pia03377

 The known specimens of this species are from the Ozark Plateau of Missouri and Arkansas (Fig. 6).

#### Bembidion rolandi

Fall, 1922

http://species-id.net/wiki/Bembidion_rolandi

##### Diagnosis.

 As discussed above, specimens of this species cannot be separated from *Bembidion ozarkense* using external characteristics, although they can be distinguished by male genitalia; see the diagnosis under *Bembidion ozarkense* for details.

Geographic distribution. Currently recorded from **Canada**: NB, NS, ON, QC; **USA**: DC, MA, MD, ME, NJ, NY, OH, PA, VA, VT, WV (Bousquet and Larochelle 1993 and R. Davidson, pers. comm.).

#### Bembidion grandiceps

Hayward, 1897

http://species-id.net/wiki/Bembidion_grandiceps

##### Diagnosis.

 Most specimens can be separated from *Bembidion fugax* and *Bembidion planum* by a combination of: broad head and pronotum; prothorax markedly constricted at base with the lateral margins just in front of the hind angle parallel, although this parallel region is much shorter than in *Bembidion rolandi* or *Bembidion ozarkense*; elytra quite flat at apex; and broad, markedly delimited frontal furrows that are clearly diverging between eyes and extended beyond the posterior supraorbital puncture.

##### Geographic distribution.

 We have seen specimens from **USA**: TX, KS, IA. Reports from the literature (Hayward 1897) of the presence of *Bembidion grandiceps* in **USA**: DC, MA, NJ, NY, and PA are likely incorrect, and may be based on *Bembidion fugax*.

#### Bembidion fugax

(LeConte, 1848)

http://species-id.net/wiki/Bembidion_fugax

##### Junior synonyms.

*Octhedromus planipennis* LeConte, 1850; *Bembidion champlaini* Casey, 1918.

##### Diagnosis.

 Most specimens can be separated from *Bembidion grandiceps* using the characters listed for that species. Also, specimens of *Bembidion fugax* have a more narrow head and pronotum and the elytral apex slopes slightly.

This species can be separated from *Bembidion planum* by the 6^th^ elytral stria being as, or nearly as, impressed behind the shoulder as the 5^th^, and the frontal furrows are prolonged and markedly diverging behind the posterior supra-orbital puncture. In addition, *Bembidion fugax* has deeper furrows with steeper sides, more polished especially at the bottom, and more sharply etched at the bottom, with the raised area between the eye and the frontal furrow being more bulbous, and shinier; *Bembidion planum* has broad, shallow furrows, sides gently sloped, more microsculpture on the slopes and especially bottoms, not sharply etched at the bottom, and with the raised area between the eye and the frontal furrow being flatter and not as shiny. Specimens tend to be darker and slightly larger than those of *Bembidion planum* and in most specimens the pronotal microsculpture is weakly impressed and obsolete on the disk.

##### Geographic distribution.

Recorded from **USA**: DC, IL, IN, MA, MD, NJ, NY, OH, PA, TN, VA, VT (Bousquet and Larochelle 1993 and R. Davidson, pers. comm.).

#### Bembidion planum

(Haldeman, 1843)

http://species-id.net/wiki/Bembidion_planum

##### Junior synonyms.

*Bembidium guexii* Chaudoir, 1868: 242; *Bembidion vulsum* Casey, 1918: 55; *Bembidion filicorne* Casey, 1918: 56.

##### Diagnosis.

 Most specimens of this species can be separated from *Bembidion grandiceps* using the characters listed for that species. Also, specimens of *Bembidion planum* have a narrower head and pronotum and the elytral apex slopes slightly.

Most specimens of this species can be separated from *Bembidion fugax* by the more weakly impressed 6^th^ compared with 5^th^ elytral stria, and the frontal furrows end just behind the posterior supra-orbital puncture and are little divergent. Specimens tend to be paler and slightly smaller than those of *Bembidion fugax*, and in most specimens have strongly-impressed microsculpture over the entire pronotum.

##### Geographic distribution.

Recorded from **Canada**: NB, NS, ON, QC; **USA**: AR, CT, DC, IA, IL, IN, KY, MA, MD, ME, MI, MN, MO, MS, NC, NH, NJ, NY, OH, PA, RI, SC, TN, VA, VT, WI, WV (Bousquet and Larochelle 1993 and Maddison, unpublished).

## Supplementary Material

XML Treatment for Bembidion ozarkense

XML Treatment for Bembidion rolandi

XML Treatment for Bembidion grandiceps

XML Treatment for Bembidion fugax

XML Treatment for Bembidion planum
